# SuRankCo: supervised ranking of contigs in de novo assemblies

**DOI:** 10.1186/s12859-015-0644-7

**Published:** 2015-07-30

**Authors:** Mathias Kuhring, Piotr Wojtek Dabrowski, Vitor C. Piro, Andreas Nitsche, Bernhard Y. Renard

**Affiliations:** 1 Research Group Bioinformatics (NG4), Robert Koch Institute, Berlin, Germany; 2 Central Administration 4 (IT), Robert Koch Institute, Berlin, Germany; 3 Centre for Biological Threats and Special Pathogens (ZBS 1), Robert Koch Institute, Berlin, Germany; 4 CAPES Foundation, Ministry of Education of Brazil, Brasília - DF, 70040-020 Brazil

**Keywords:** De novo assembly, Genome assembly, Next generation sequencing, Contigs, Quality control, Machine learning, Random forest

## Abstract

**Background:**

Evaluating the quality and reliability of a de novo assembly and of single contigs in particular is challenging since commonly a ground truth is not readily available and numerous factors may influence results. Currently available procedures provide assembly scores but lack a comparative quality ranking of contigs within an assembly.

**Results:**

We present SuRankCo, which relies on a machine learning approach to predict quality scores for contigs and to enable the ranking of contigs within an assembly. The result is a sorted contig set which allows selective contig usage in downstream analysis. Benchmarking on datasets with known ground truth shows promising sensitivity and specificity and favorable comparison to existing methodology.

**Conclusions:**

SuRankCo analyzes the reliability of de novo assemblies on the contig level and thereby allows quality control and ranking prior to further downstream and validation experiments.

**Electronic supplementary material:**

The online version of this article (doi:10.1186/s12859-015-0644-7) contains supplementary material, which is available to authorized users.

## Background

In contrast to mapping procedures, de novo assembled sequences lack the direct comparison to a reference genome and thus have no ground truth-based quality control readily available. Commonly, evaluation of de novo assemblies and their contigs is based on single metrics (such as the N50) and their individual interpretation [1] or on evaluations of accumulated metrics or mis-assembly features [2–5]. Several methods and tools were released lately that introduced a new degree of quality detail on a nucleotide level, such as ALE [6], CGAL [7], LAP [8] or REAPR [9]. They provide log-likelihoods based on probabilistic assumptions to allow quality comparison between different assemblies.

In this contribution, we focus on the aspect of quality control within a de novo assembly. We introduce a machine learning based method to evaluate and rank contigs within a single de novo assembly, called SuRankCo (Supervised Ranking of Contigs). The method takes advantage of data already generated in related sequencing experiments. It allows the selection of a suitable subset of contigs for subsequent processing and analysis.

In general, not every contig can be assumed to be error-free and it may save time and resources to re-strict downstream analysis to reliable information. In doing so, for instance, conflicts in finishing procedures may be prevented [10, 11], expensive validation experiments can focus on contigs of sufficient quality [12, 13] and ambiguities in derived gene annotations may be explained by contig quality [14].

Surankco ranks contigs by their quality and can help in identifying the error source by the various scores it produces. However, it is outside of the scope of this manuscript to improve low-ranking contigs and repair their errors. There are other strategies and tools which are applicable, e.g. the integration of different assembler types with non-overlapping error profiles [15], the application of error correcting tools for the reads [16], or the critical visual inspection and manual correction [11].

The main idea of SuRankCo is to rely on knowledge generated from contigs from sequencing experiments of related organisms for which a genome reference is available. Aligning these contigs to the reference yields scores which can be used as targets for a machine learning approach. Contigs from a new assembly can then be examined and classified with respect to the learnt target scores based on different features.

In the following, we introduce the methodology and implementation of SuRankCo, evaluate it on bacterial de novo genome assemblies and compare to ALE as an existing and related method.

## Implementation

**Fig. 1:**
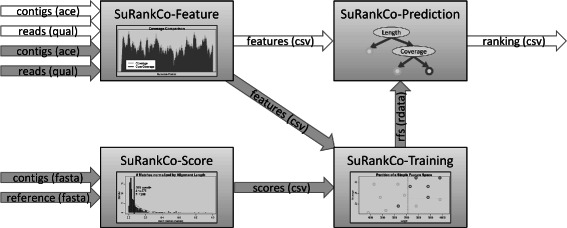
Modularization and workflow of SuRankCo. The four modules of SuRankCo allow two workflows, training and prediction, indicated by grey and white arrows, respectively

SuRankCo is divided into four modules (illustrated in Fig. 1), including the extraction of contig features, the calculation of alignments and single scores, the training based on features and the prediction of single scores based on features to build the ranking. These modules can be combined to either perform training or prediction. In addition, intermediate data such as the features, single scores or trained classifiers can be examined or used within other applications.

### SuRankCo-feature

Information on characteristics of contigs from a de novo assembly are extracted by the SuRankCo-Feature module. These features include common characteristics such as length (unpadded and padded), coverage, quality values, read counts, read lengths and read quality values. Additional features were developed, including core coverage, coverage confirmation and coverage drops. For a full list of features and descriptions refer to the supplementary material. SuRankCo-Feature accepts assemblies either as a pair of ace and fastq files or fasta and sam/bam files, respectively.

### SuRankCo-score

Training contigs are scored by comparison to a corresponding reference genome sequence. The SuRankCo-Score module utilizes BLAT [17] and accompanying tools to build alignments. Next, several single scores are calculated for each contig based on these alignments. Some scores are computed for each contig as a whole and some for certain critical areas such as the contig ends. Additionally, some scores are varied by introducing different normalizations, for instance based on contig or alignment length. A full list and descriptions of the single scores is given in the supplementary material.

### SuRankCo-training

The classification of contigs in SuRankCo is performed using a random forest classifier [18]. Here, we rely on a random forest classifier as it adapts to different scenarios without the need for parameter tuning, can handle discrete and continuous input and can also uncover non-linear relationships. The training of the random forests is preceded by a separation of each single score into two classes to allow for binary classification using quantiles of fitted exponential distributions. Alternatively, a manual adjustment is possible based on histograms provided by the SuRankCo-Score module. A detailed description is given in the supplementary material. Finally, the SuRankCo-Training module uses contig features and the transformed single scores to train a classification random forest for each score.

### SuRankCo-prediction

The SuRankCo-Prediction module estimates single score classes from contigs and their respective features by using the previously trained random forests. Different estimates are aggregated in a voting procedure to provide a final SuRankCo contig score. It is defined as $\sum _{i=1}^{|S|} S_{i} \times P_{i} \label {eq:01}$ where *S*_*i*_ is the *i* ^*th*^ *s* single score classification (0 or 1) and *P*_*i*_ denotes the probability of *S*_*i*_ being classified to that class, which is provided by the random forests. The SuRankCo contig score determines the final position in the ranking of the contigs.

## Results and discussion

### Experiments

We evaluate the application and classification quality of SuRankCo by using various publicly available genome sequencing data sets. In the first experiment, we apply SuRankCo on the well-studied *Escherichia coli* strain K-12, substrain MG1655 [19] and compare to ALE as an existing and related method. We constructed four de novo assemblies of Illumina Genome Analyzer II reads from the NCBI Sequence Read Archive (SRA), three for training and one for prediction and evaluation (accession numbers are provided in the supplementary material). The training and the evaluation of the predictions make use of an established high quality reference [NCBI:NC_000913.3]. However, it should be noted that using the same organism for training and prediction is an artificial application as a proof-of-principle. Details on the data preparation are given in the supplementary material.

We calculated the classification quality for each single score by comparing predicted classes versus real classes. As additional validation with ground truth data, we compared the ranking based on the SuRankCo contig scores to the percentage identity (pIdent) of Blast hits in the current NCBI *E. coli* taxon [taxid:562], assuming that more reliable contigs should show better identity values.

Current methods for quality control in de novo assemblies do not score individual contigs, but rather focus on comparing complete assemblies. In order to still provide a meaningful comparison, we counted potential contig errors based on ALE sub-scores. Therefore, we manually evaluated the sub-scores and defined error thresholds (see Additional file 1: Figure S1). Sub-scores below their corresponding thresholds are counted as error and errors are summed per contig over all positions. For the *E. coli* prediction data set, these ALE contig scores were then compared to the Blast pIdent values in the same way as the SuRankCo contig scores. More details on the application of ALE are given in the supplementary material.

To demonstrate the applicability for different organisms and assemblers, we applied SuRankCo on the staggered mock community of the Human Microbiome Project [20] and the bacteria assemblies of the GAGE study [15]. We used three different settings for the mock community: (i) a metagenomics assembly, (ii) an organism specific assembly with different assemblers, and (iii) a combined training on assemblies by various assemblers. For (i), we constructed a meta-assembly of the complete community. We then assigned the resulting contigs to the respective organisms and then randomly divided the set of organisms in the community into a training and a prediction group. For (ii), we extracted all reads for each organism by a reference mapping procedure to have single organism sequencing data with identical technical origin. Each organism was then assembled separately using the assemblers Mira [21], SOAPdenovo [22] and Velvet [23]. Training and prediction was performed for each assembler separately with a separation of organisms as in the metagenomics assembly experiment. For (iii), the assemblies of the different assemblers in (ii) were merged to provide a training and prediction data set across all organisms and assemblers. Details on the data preparation are given in the supplementary material.

For the SuRankCo analyses of the GAGE bacteria, we made use of the assemblies, reads, and genomes provided for *Staphylococcus aureus* and *Rhodobacter sphaeroides*. In particular, we used the *S. aureus* assemblies for training and *R. sphaeroides* for prediction. We used two different settings for the GAGE assemblies: (i) an assembler specific training, and (ii) a combined training on assemblies by various assemblers. For (i), training and prediction was performed for each assembler used in the GAGE study separately. For (ii), the assemblies of the different assemblers were merged to provide a training data set across all assemblers. Details on the data preparation are given in the supplementary material.

To evaluate the mock and GAGE experiments, we compared the SuRankCo score rankings to Blast hits of contigs mapped against the corresponding known reference genomes. In particular, we calculate a contig evaluation score by forming the harmonic mean between the Blast pIdent and the Blast query coverage (qcovhsp). We then assigned the contigs based on the ground truth into a low-quality and a high-quality group and evaluated the performance of SuRankCo by ROC curves.

In addition, we compared the SuRankCo results of the GAGE assemblies to the corresponding GAGE evaluation metrics including contig number, errors, N50, and corrected N50. We calculated mean values of final SuRankCo contig scores per assembler in order to enable ranking based comparisons assuming a correlation between SuRankCo score distribution order of the different assemblies and their corresponding GAGE evaluation metrics.

### Comparative evaluation

**Fig. 2:**
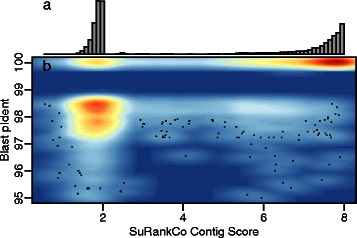
Evaluation of the SuRankCo rankings on the *E. coli* test data. **a** shows the distribution of SuRankCo contig scores. They form two clusters based on the high correlation of target scores in this data set. Clusters are skewed due to classification probabilities incorporated into the SuRankCo contig scores. **b** shows a scatterplot comparison of the ranking and the pIdent of Blast matches against the *E. coli* taxon. High and low density areas are indicated in red and blue, respectively. Data points below 95 % pIdent are not shown to improve the scaling (25 of 11336)

The *E. coli* experiment illustrates three key characteristics of the single scores. First, the contigs used in training show good quality in their alignments to the reference sequence. Thus, they feature low variance in the single score distributions. Second, these variances are still sufficient to allow an automated separation into two classes (see Additional file 1: Figure S2). Third, a successful prediction can be made with a low number of false positives and false negatives in the test data (see Additional file 1: Figure S3). Further, the validity of the SuRankCo contig score is supported by a comparison to the percentage identity of the corresponding Blast hits (Fig. 2 b) with Pearson and Spearman correlation coefficients of 0.77 and 0.72, respectively.

**Fig. 3:**
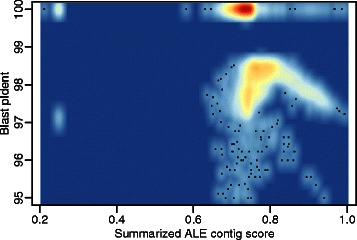
Evaluation of ALE contig scores of E. coli test data. The figure shows a scatterplot comparison of the ALE contig scores and the pIdent of Blast matches against the *E. coli* taxon. High and low density areas are indicated in red and blue, respectively. The ALE contig scores are shown in reversed order to allow a simpler comparison to Fig. 2. Data points below 95 % pIdent are not shown to improve the scaling (25 of 11336)

Figure 3 shows a comparison of ALE contigs scores and Blast pIdent values. In addition, the comparative evaluation results for SuRankCo and ALE on contigs of varying length are shown in Table 1. Correlations between Blast pIdent values and SuRankCo contig scores are generally higher than correlations between Blast pIdent values and ALE contig scores, independent of whether Spearman or Pearson correlation is used and how long contigs are. However, it should be noted that ALE was applied here outside its regular scope and results should by no means be interpreted as general criticism of the tool. To the contrary, differences in the performance between SuRankCo scores and ALE scores only emphasize the differences regarding their approaches and objectives. The fact that ALE does not provide contigs scores directly further supports this observation.

**Table 1:** Comparative evaluation of SuRankCo and ALE

Score	Contig Length	Cor _*Pearson*_	Cor _*Spearman*_
SuRankCo	all	0.77	0.72
ALE	all	0.35	0.49
SuRankCo	≤*Q*_0.1_	0.58	0.55
ALE	≤*Q*_0.1_	0.16	0.37
SuRankCo	≥*Q*_0.9_	0.75	0.68
ALE	≥*Q*_0.9_	0.19	0.12

The table shows the Spearman and Pearson correlations of SuRankCo and ALE contig scores to the percentage identity of corresponding Blast hits. The correlations are calculated for all contigs as well as separately for short contigs (with lengths below the 10% quantile) and long contigs (with lengths above the 90% quantile)

For SuRankCo, a high correlation between the single scores is notable in the *E. coli* experiment (as shown in Additional file 1: Figure S6). However, correlated scores do not corrupt the predictions, but favor clustering of contigs within the ranking rather than a more uniform distribution (compare Fig. 2 a). In general, contig scores may be less correlated and thus provide a wider distribution of SuRankCo contig scores in the ranking as shown for the data of the metagenomics mock community experiment in Additional file 1: Figure S7. In addition, the variety of SuRankCo contig scores enables a broader integration and indentification of common assembly error types (see Additional file 1: Table S5 and S6).

**Fig. 4:**
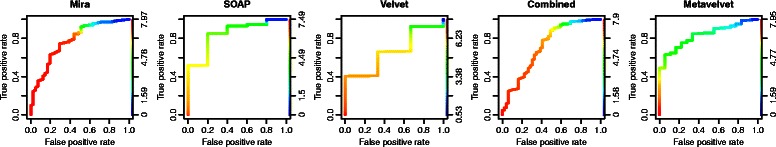
Evaluation of the SuRankCo predictions of the mock community test data. Each plot illustrates a ROC curve of the contig evaluation score grouping in contrast to a varying grouping of the SuRankCo scores. Thereby, the changing color of the graph represents the changing threshold for the SuRankCo score grouping. Here, the predictions for the different organisms in the test group are combined to feature ROC curves of specific, combined and meta-assemblies

The mock experiments allow a detailed view on parameters influencing SuRankCo results. Altogether, results indicate good prediction with regard to true positive rates (TPR) and false positive rates (FPR) (see Fig. 4). However, some exceptions can be observed on the organism and on assembler level as exemplified in Additional file 1: Figure S4. In general, merging the training data from various assemblers does not improve on individual assembler results, but rather has negative effects. This indicates that there are assembler specific error types that can be learnt with SuRankCo. Comparing assembler results, the evaluation of Velvet assemblies performs poorly in contrast to the other assemblers. However, for Velvet we observed the lowest number of contigs with low quality based on the Blast generated ground truth. This indicates that the performance of SuRankCo decreases for assemblies of very high quality since there is only few variance left for proper training or prediction. For organisms, we note that comparatively poor results are obtained for *S. epidermidis*, in particular for Mira, Metavelvet and the combined assemblers, although an apparently closely related organism (*S. aureus*) is present in the training data. However, examining the relation of mock organisms based on sequencing data reveals low similarities in general (as shown in Additional file 1: Table S7).

**Fig. 5:**
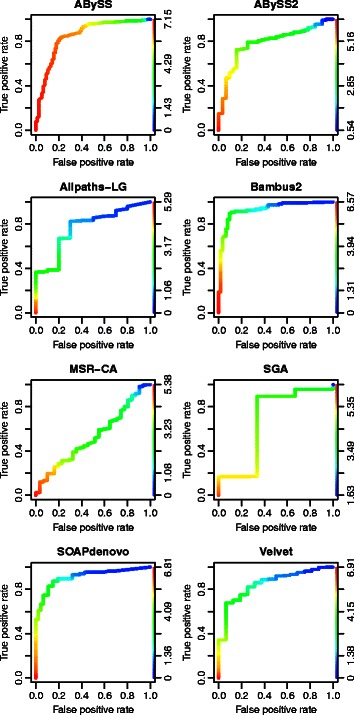
Evaluation of the SuRankCo predictions of the GAGE assemblies. Each plot illustrates a ROC curve of the contig evaluation score grouping in contrast to a varying grouping of the SuRankCo scores. Thereby, the changing color of the graph represents the changing threshold for the SuRankCo score grouping. Here, one ROC curve represents the evaluation of *R. sphaeroidis* assemblies classified by the combined training classifier

Similar to the mock experiments, the GAGE experiments result in overall accurate predictions as illustrated by the ROC curves in Fig. 5. However, few assemblies yield low prediction power including MSR-CA and SGA. The comparably low error rate in these two assemblies (as shown in Table 2) supports the conclusion that the performance of SuRankCo decreases for assemblies with very few errors. Since SuRankCo is a learning based approach, it requires also negative examples containing errors in the assemblies. If these are missing, artifacts may arise more frequently. In summary, assemblies which provide a few, but potentially error-prone contigs may benefit more from SuRankCo than assemblers with a high number of short, but error-free contigs.

**Table 2:** Contig metric values of *R. sphaeroides* assemblies as provided by the GAGE study

Assembler	Num	N50	Errors	N50corr
ABySS	1915	5.9	76	4.2
ALLPATHS-LG	204	42.5	49	34.4
Bambus2	177	93.2	373	12.8
MSR-CA	395	22.1	52	19.1
SGA	3067	4.5	12	2.9
SOAPdenovo	204	131.7	422	14.3
Velvet	583	15.7	43	14.5

Note, ABySS2 metric values were not available

**Fig. 6:**
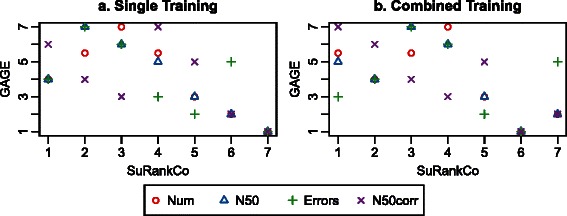
Scatterplot of the SuRankCo score mean ranks and GAGE metric ranks. The figure shows scattorplots of ranks for GAGE assemblies of *R. sphaeroidis* based on the SuRankCo score means vs. each GAGE metric including contig number, errors, N50, and corrected N50. **a** features SuRankCo score mean ranks based on assembler specific trained classifier and **b** based on the combined trained classifier, respectively. To improve visualization, the contig number ranks have been inverted since they are the only ones yielding positive correlation

In contrast to the mock experiment, on average there is no significant difference between predictions based on assembler specific (Additional file 1: Figure S5) or combined training (Fig. 5). However, the correlation of SuRankCo score means with the GAGE error metric shows a significant decrease from assembler specific to combined training based predictions (Table 3). Again, this indicates that there are assembler specific characteristics that can only be learnt and discriminated by separate training. Apart from that, the comparison of SuRankCo and GAGE yields good rank correlations with values of up to 0.85 as shown in Table 3 and Fig. 6 for both, assembler specific and combined training and prediction. Therefore, based on independent ground truth data, the correlations indicate that SuRankCo infers the relationship of different assemblies in terms of quality, even if trained separately. Nonetheless, as also indicated by the diversity of the metrics in the GAGE study itself, it is difficult to perfectly represent the quality of assemblies in few scores. Thus, it cannot be expected to observe a direct one-to-one correspondence of SuRankCo scores with single GAGE metrics. At the same time, it should be noted that SuRankCo was developed to score individual contigs and that the overall ranking of assemblies by their mean ranking score - while well correlated with the metrics in the GAGE study - is not its standard usage.

In classic assembly metrics such as the N50, a high value is placed on obtaining longer contig scores. However, it has been frequently noted that longer contig scores do not necessarily coincide with higher contig quality [24]. SuRankCo scores are evaluated with regard to the identity and query coverage of the reference genome. Increasing values in these metrics may correlate with longer contigs, but are by no means ensured and rather focus on the number of matches and mismatches.

**Table 3:** Comparative evaluation of SuRankCo and GAGE

	Num	N50	Errors	N50corr
Specific Training	0.7208	-0.7857	-0.6071	-0.6071
Combined Training	0.6847	-0.6786	-0.1786	-0.8571

The table shows the Spearman correlations between assembly ranks based on SuRankCo score means and GAGE metrics for *R. sphaeroides* assemblies. Correlations are calculated for SuRankCo ranks based on assembler specific trained classifier as well as combined trained classifier

Overall, several factors may influence the assembly of contigs significantly and thereby also influence the performance of SuRankCo. These include for instance sequencing parameters such as coverage and read length, sequencer error profiles, organism relationships, biases such as GC content and characteristics of read processing algorithms such as these used for de novo assembly. Thus, SuRankCo is mainly designed with a focus on stable workflows applied within a lab. SuRankCo has been mainly developed for and tested on microbial genomes, however, there is no theoretical limitation which should restrict the application to other genomes.

## Conclusions

We introduced SuRankCo as a tool for a learning-based quality prediction and ranking of contigs within a de novo assembly. To take full advantage of the machine learning approach and for optimal performance, training and test data have to be similar in their key characteristics. In our benchmark, we observe promising results in terms of sensitivity and specificity and favorable comparison to existing methodology. We foresee practical application in ranking contigs for downstream analyses.

## Availability and requirements

**Project name:** SuRankCo**Project home page:** http://sourceforge.net/projects/surankco/ **Operating systems:** Linux, OS X **Programming language:** Java, R **Other requirements:** Java 7, GNU R 3 including packages (optparse, MASS, randomForest), Blat including pslPretty **License:** BSD License **Any restrictions to use by non-academics:** non

## Additional file

Additional file 1 **Supplementary Material for “SuRankCo: Supervised Ranking of Contigs in de novo Assemblies”.** Provides details on contig features, contig scores, training class definitions and experiment preparation as well as additional result figures and tables.
