# Supplementary material for: SuRankCo: supervised ranking of contigs in de novo assemblies
Source: BMC Bioinformatics. 2015 Jul 30;16:240. doi: 10.1186/s12859-015-0644-7 (PMC4520199; doi:10.1186/s12859-015-0644-7)
Supplement: Supplementary file 1 — Supplementary Material for “SuRankCo: Supervised Ranking of Contigs in de novo Assemblies”. Provides details on contig features, contig scores, training class definitions and experiment preparation as well as additional result figures and tables. [file 12859_2015_644_MOESM1_ESM.pdf]

Supplementary Material for "SuRankCo: Supervised Ranking of Contigs in de novo Assemblies"

Mathias Kuhring[1], Piotr Wojtek Dabrowski[2,3],
Vitor C. Piro[1], Andreas Nitsche[3] and Bernhard Y. Renard[1,*]

[1]Research Group Bioinformatics (NG4), [2]Central Administration 4 (IT),
[3]Centre for Biological Threats and Special Pathogens (ZBS 1),
Robert Koch Institute, Berlin, Germany

S1 Contig Features

The features applied by SuRankCo are listed in Supplementary Table 1 with name, description and variants.

Some features are based on single values and thus have no variants. Other features consisting of several values per contig (such as coverage, read count etc.) are summarized by six common variants to describe their statistical distribution. This includes the mean, the standard deviation (sd), the median, the median absolute deviation (mad), the minimum and the maximum.

Further variants are noted in the description as *additional variants*, if appropriate.

Table 1: Contig Features

Name	Description	Variants
**Length**	Unpadded length, i.e. length of the final contig.	
**Base Count**	Padded length, i.e. length of the consensus of a read alignment, including potential gaps introduced by inserts in reads which are not consistent with the majority.	
**Base Segment Count**	Number of continuous gapless segments in the padded contig.	
**Read Count**	Number of reads contributing to the contig.	
**Read Complement Fraction**	The fraction of complement reads in the total number of reads contributing to the contig.	
**N50 Relation**	Relation between contig length and N50.	
**Estimated Genome Size**	The expected genome size (EGS), either indicated by a parameter or estimated as the sum of contig lengths.	
**Genome Relation**	Relation between the contig length and the estimated genome size.	
**Contig Qualities**	Pooled base-wise quality values of the contig as provided from assemblers (e.g. in Ace files).	✓
**Read Qualities**	Pooled base-wise quality values of the reads as provided from base-callers (e.g. in Sff files).	✓

*to whom correspondence should be addressed

**Read Length**	Pooled original lengths of the reads.	✓
**Read Length Padded**	Pooled padded lengths, i.e. lengths of the reads in the alignment, including potential gaps introduced by deletions in other reads which are not consistent with the majority (compare Base Count).	✓
**Read Length Quotient**	Relation between original read length and padded read length (for all six variants).	
**Read Length Clipped**	Pooled lengths of clipped reads, i.e. the lengths of the padded read parts which are actually used and thus contribute to the contig (for instance, some read ends do not).	✓
**GC-Content**	The fraction of GC-content in the contig.	
**Coverage**	Pooled number of reads contributing to each position in the contig.<br>*Additional variants*: Contig ends coverage is reported in addition, with end size equal to read length mean.	✓
**Core Coverage**	Pooled number of reads contributing to each position in the contig with the same nucleotide as the one selected for the consensus.<br>*Additional variants*: Contig ends core coverage is reported in addition, with end size equal to read length mean.	✓
**Base Confirmation**	Significance of the core coverage in contrast to the coverage per position, tested with a binomial test with $k = \textit{core coverage}$, $n = \textit{coverage}$ and $p = 0.98$. $p = 1 - \textit{error rate}$, where error rate denotes the average sequencing error. With an error rate of 2% the expectation of reads contributing the same correct nucleotide to each position is therefor 98%.<br>*Additional variants*: Contig ends base confirmation is reported in addition, with end size equal to read length mean.	✓
**Coverage Comparison**	Coverage comparison within an assembly represented by the relation of the contig coverage to the mean coverage of all contigs in the assembly.<br>*Additional variants*: Contig ends coverage comparisons are reported in addition, with end size equal to read length mean.	
**Coverage Curve Drops**	Coverage curve drops indicate local minima in the coverage of a contig with a value of less than 25% and 50% in contrast to their adjacent maxima within a fixed window size w. The coverage is preprocessed with a sliding window smoothing with window size w which is chosen as the mean read length of a contig. The number of drops is reported normalized by the contig length as well as the biggest drop, i.e. maximal difference between a minima and its smaller adjacent maxima.	

**K-mer Uniqueness Global**	Number of K-mers unique in a contig in contrast to other contigs within the assembly normalized by the contig length (since longer contigs comprise more unique K-mers by chance). K-mers are extracted with a size of 8, and only K-mers containing standard nucleotide symbols (i.e. A,C,G and T) are considered.
**K-mer Uniqueness Ends**	Number of K-mers unique in a contig end in contrast to both ends of other contigs within the assembly. Reported as minimal and maximal K-mer uniqueness to avoid implicit orientation of the contig. The read length mean of a contig is chosen as end size.

S2 Contig Scores

Supplementary Table 2 provides an overview of the single contig scores calculated by SuRankCo. The scores are either based on match counts or error counts (edit distance, including mismatches and gaps) of contig-reference alignments.

Table 2: Contig Scores

Name	Description & Motivation
General Scores	Account for mismatches/errors in general as well as insertions and deletions to the contig (Normed Match Count 1 resp. Normed Match Count 2). These scores provide a basic penalization for small errors in general.
**Normed Match Count 1**	Number of alignment matches normalized by the contig length.
**Normed Match Count 2**	Number of alignment matches normalized by the alignment length.
**Normed Error Count 1**	The edit distance respectively error count normalized by the contig length.
Large Error Scores	Account for very large errors or unstable regions which might originate from mis-joins or badly sequenced/covered regions, resp. While small errors are only considered by the General Scores, critical large errors are additionally penalized hereby.
**Max. Contiguous Error**	The largest contiguous stretch of alignment errors normalized by the contig length.
**Max. Region Error**	The largest number of alignment errors in a fixed region size (100 bp).
End Scores	Similar to Large Error Scores but applied to contig ends only. Errors in this region are rather critical for subsequent applications (e.g. for scaffolding) are therefore additionally penalized.
**Max. End Error Stretch**	Largest stretch of errors right at the contig ends (unfixed length) normalized by the contig length.
**Max. End Error Count**	The largest number of alignment errors in the ends (fixed length of 100 bp).
Other Scores	Additionally account for insertions and critical mis-joins.
**Normed Contig Length**	Relation of contig length to alignment length.

S3 Training Class Definitions

Each contig score applied by SuRankCo is separated into two classes to allow for binary classification. The separation into the two classes can be either set manually or automatically by fitting exponential distributions. A threshold selection may be supported by histograms provided by the SuRankCo-Score module (as shown in Supplementary Figure 2).

The automatic exponential fitting makes use of the MASS R package (Venables and Ripley, 2002). It fits an exponential distribution to each single score distribution of the training contigs. Finally, a certain quantile of a fit is considered as the threshold for the score class separation. The selection of a quantile should be based on the concrete training data and be adjusted accordingly. However, 25% yielded a good separation for the *E. coli* contigs. See Supplementary Figure 2 for examples.

S4 Experiment Preparation

S4.1 E. Coli

To demonstrate the usage of SuRankCo, we chose four next-generation sequencing experiments available in the NCBI Sequence Read Archive (SRA) (Wheeler *et al.*, 2008). The reads were all sequenced with an Illumina Genome Analyzer II. Additional properties are listed in Supplementary Table 3.

Table 3: SRA Experiments

**Experiment ID**	**# of Spots**	**# of Bases**
SRR400617	14,299,251	514.8M
SRR400618	13,539,459	487.4M
SRR400619	16,720,568	601.9M
SRR400620	16,359,717	588.9M

The reads were assembled using Mira (Chevreux *et al.*, 1999) with basic settings. A sample configuration for one experiment is provided in listing 1. Finally, the four resulting assemblies were randomly divided into three training assemblies (SRR400617, SRR400618 and SRR400619) and one test assembly (SRR400620).

Listing 1: Mira Configuration Manuscript

```
project = SRR400617
job = genome,denovo,accurate

readgroup = IlluminaReads
data = SRR400617.fastq
technology = solexa

parameters = -GE:not=10 -NW:cmrnl=warn -OUT:ora=on
```

S4.2 ALE

To compare the SuRankCo results of the E. coli experiment to ALE, the reads of the prediction data (SRR400620) were mapped against the corresponding contigs using Bowtie2 with default settings. Thereby, ambiguous reads where assigned according to the best alignment. The resulting sam file were sorted and, together with the contigs provided to ALE.

Since ALE does not provide a score per contig, ALE sub-scores were transformed to error counts and summed up per contig. For each ALE sub score, a histogram over all contigs and positions

was created to manually choose a threshold (as shown in Supplementary Figure 1). Each contig position below the threshold of a sub-score is counted as a potential error. The counts were summed for each contigs and normalized by the contigs length and the total number of sub-scores.

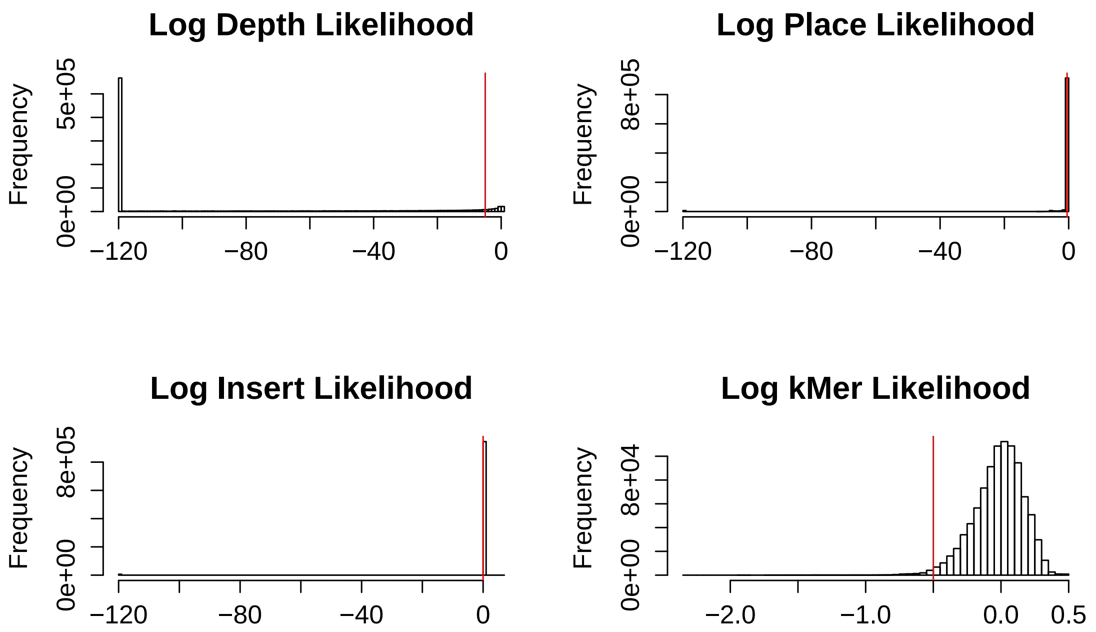

Figure 1: Histogram of ALE sub-scores. For each sub-score, histogram were constructed over all contigs and position. Thresholds were set manually to -5, -0.5, 0 and -0.5 for the ALE sub-scores Log Depth Likelihood, Log Place Likelihood, Log Insert Likelihood and Log kMer Likelihood, respectivly.

S4.3 Mock Community

To demonstrate the usage of SuRankCo in conjunction with several organisms and assemblers, we make use of the staggered mock community of the Human Microbiome Project. The data set is available in the NCBI Sequence Read Archive [SRA:SRR172903] with a total number of 7,932,819 reads and 595M bases. Organisms in the mock community are represented in Table 4 as well as the reference sequences used to classify reads and contigs and for evaluation. The mock data is evaluated in three different settings, a meta-assembly, single organism assemblies and a merged evaluation of single organism assemblies of different assemblers.

S4.3.1 Mock Meta-Assembly

The meta-assembly of the mock community is constructed using MetaVelvet (Namiki *et al.*, 2012) with kmer size 31 and no scaffolding. The resulting contigs are assigned to organisms by using Blast (Altschul *et al.*, 1990) against all reference sequences and selecting the best hits according to the e-value. For training and prediction of SuRankCo scores, the organisms are randomly divided into two equal groups. The grouping and number of assigned contigs is depicted in Table 4. The MetaVelvet output is converted to ace files using AMOS (Treangen *et al.*, 2011).

S4.3.2 Mock Single Assembly

For the single organism approach, the mock reads are mapped against all references and thereby assigned to organisms by using Bowtie2 (Langmead and Salzberg, 2012) with default settings. We selected all organisms with sufficient coverage for the following assemblies, including *E. coli* ($\sim$ 9x), *M. smithii* ($\sim$ 11x), *R. sphaeroides* ($\sim$ 30x), *S. aureus* ($\sim$ 38x), *S. epidermidis* ($\sim$ 35x) and *S. mutans* ($\sim$ 20x). This selection agrees with organisms comprising a suitable amount of contigs in the meta-assembly as shown in Table 4. The reads for each organism are then assembled separately with Mira (Chevreux *et al.*, 1999), Soap (Luo *et al.*, 2012) and Velvet (Zerbino and Birney, 2008) with default settings, except in the following cases. For Soap and Velvet assemblies are constructed over a range of kmers from 1 to 75 and for each organism the assembly with the highest N50 is selected for further analysis. Since not all assemblers used here provide alignment information but contig sequences only, the corresponding reads are remapped to the assemblies by using Bowtie2 with default settings to produce sam files as input for SuRankCo. For training and prediction, organisms are assigned to the same groups as for the meta-assemblies.

S4.3.3 Mock Single Assembly Merged

For the third evaluation, the single organism assemblies from Mira, Soap and Velvet are merged into combined datasets. Thus the training set and the prediction set consist each of assemblies of three organisms from three assemblers.

In general, SuRankCo is used with default settings for all mock experiments. However, contigs are filtered for a minimum size of 350 bases, since commonly no valuable information such as genes are expected to be covered by shorter sequences.

S4.4 GAGE Study

To further demonstrate the usage of SuRankCo in conjunction with several assemblers, we make use of the bacterial assemblies provided by the GAGE study. We evaluate all available assemblies of *Staphylococcus aureus* and *Rhodobacter sphaeroides* including ABySS, ABySS2, Allpaths-LG, Bambus2, MSR-CA, SGA, SOAPdenovo, Velvet. However, the CABOG assembly of *R. sphaeroides* could not be evaluated since there is no CABOG assembly of *S. aureus* available.

Since none of the GAGE assemblies provide alignment information but contig sequences only, the corresponding reads are remapped to the assemblies by using Bowtie2 with default settings to produce sam files as input for SuRankCo. For each assembly, we used either the original read set or a corrected read set in accordance with the GAGE supplementary material.

The GAGE bacteria assemblies are evaluated in two different settings including the evaluation of single assemblies and a merged evaluation of the assemblies of different assemblers. In both settings *S. aureus* has been used for training and *R. sphaeroides* for prediction. In the first setting, each assembly of *R. sphaeroides* is evaluated by training SuRankCo on the corresponding assemblies of *S. aureus* from the same assembler. For the second evaluation, the assemblies of *S. aureus* are merged into a combined training datasets. Then, each *R. sphaeroides* assembly is evaluated based on this merged training. SuRankCo is used with default settings for all GAGE experiments. However, contigs are filtered for a minimum size of 350 bases, since commonly no valuable information such as genes are expected to be covered by shorter sequences.

S5 Additional Result Figures

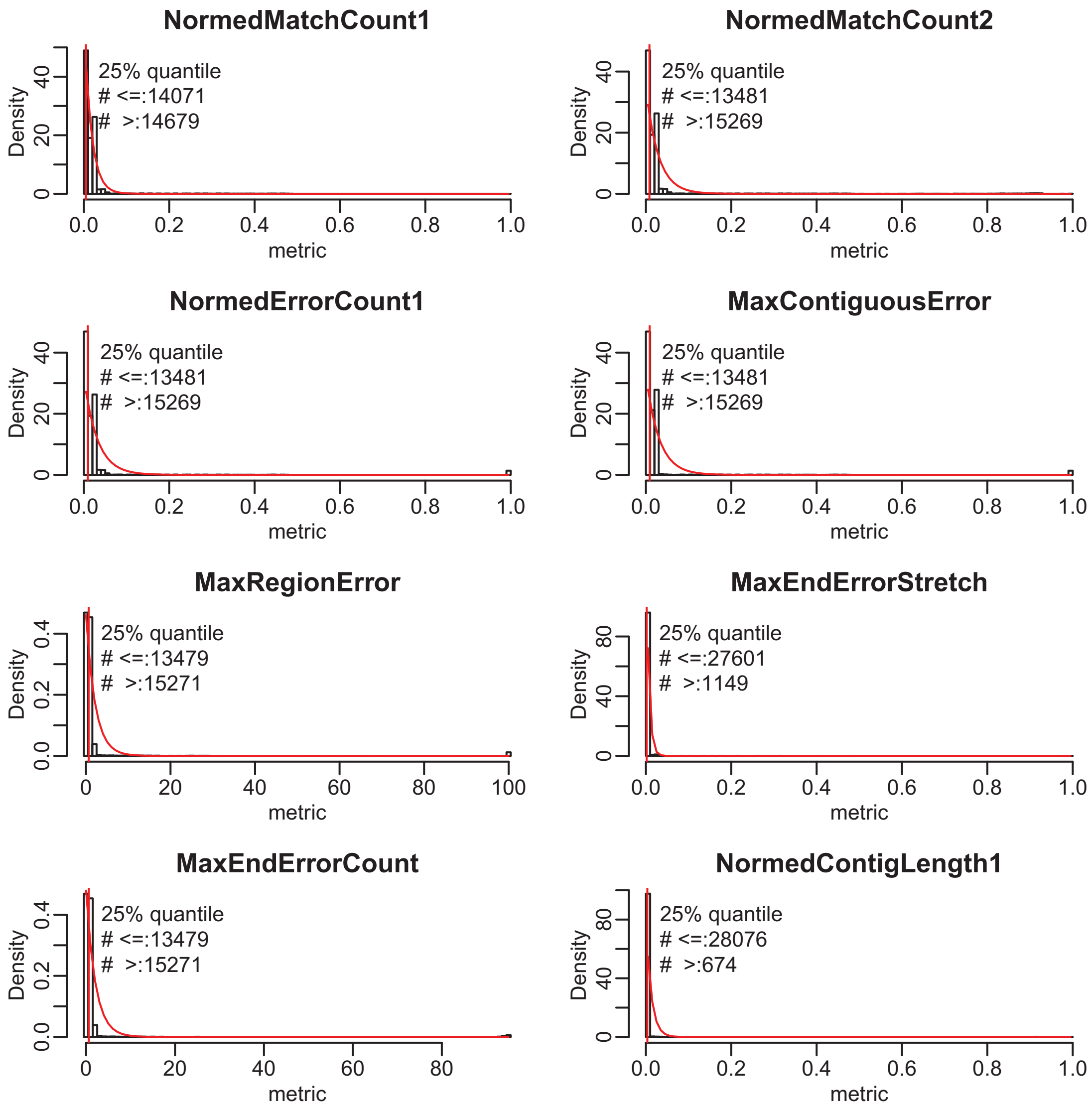

Figure 2: Histograms of single contig scores and exponential distribution fittings. For each single score, histograms (in black) are produced from the contigs of the training data. To further support the threshold selection, exponential distributions (in red) are fitted to each score. Finally, for the *E. coli* data 25% quantiles are selected as thresholds (vertical red lines). The numbers inside each plot indicate the amount of contigs below and above the threshold. The score ranges of Normed Match Count 1, Normed Match Count 2 and Normed Contig Length 1 are inverted to enable exponential fittings.

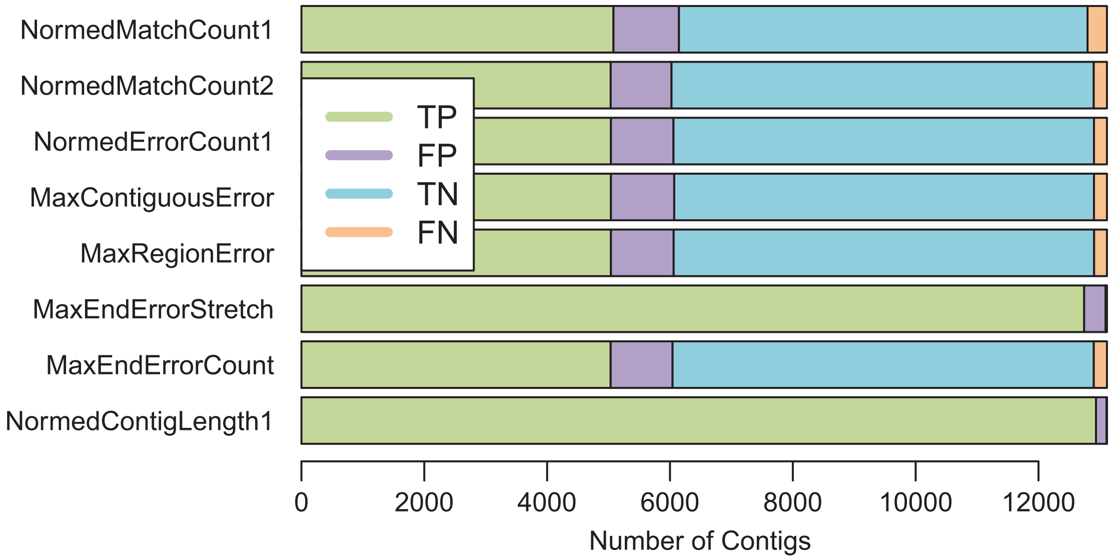

Figure 3: Classification metrics. For each score, the proportions of correct and incorrect classifications are indicated by true positives (TP), false positives (FP), true negatives (TN) and false negatives (FN). Most scores show satisfying numbers of TPs and TNs. However, the scores Max End Error Stretch and Normed Contig Length 1 have mainly true TPs, some FP and almost no TNs and FNs. This is due to the very low variance in the corresponding distributions of the training contigs (see Supplementary Figure 2 for comparisons).

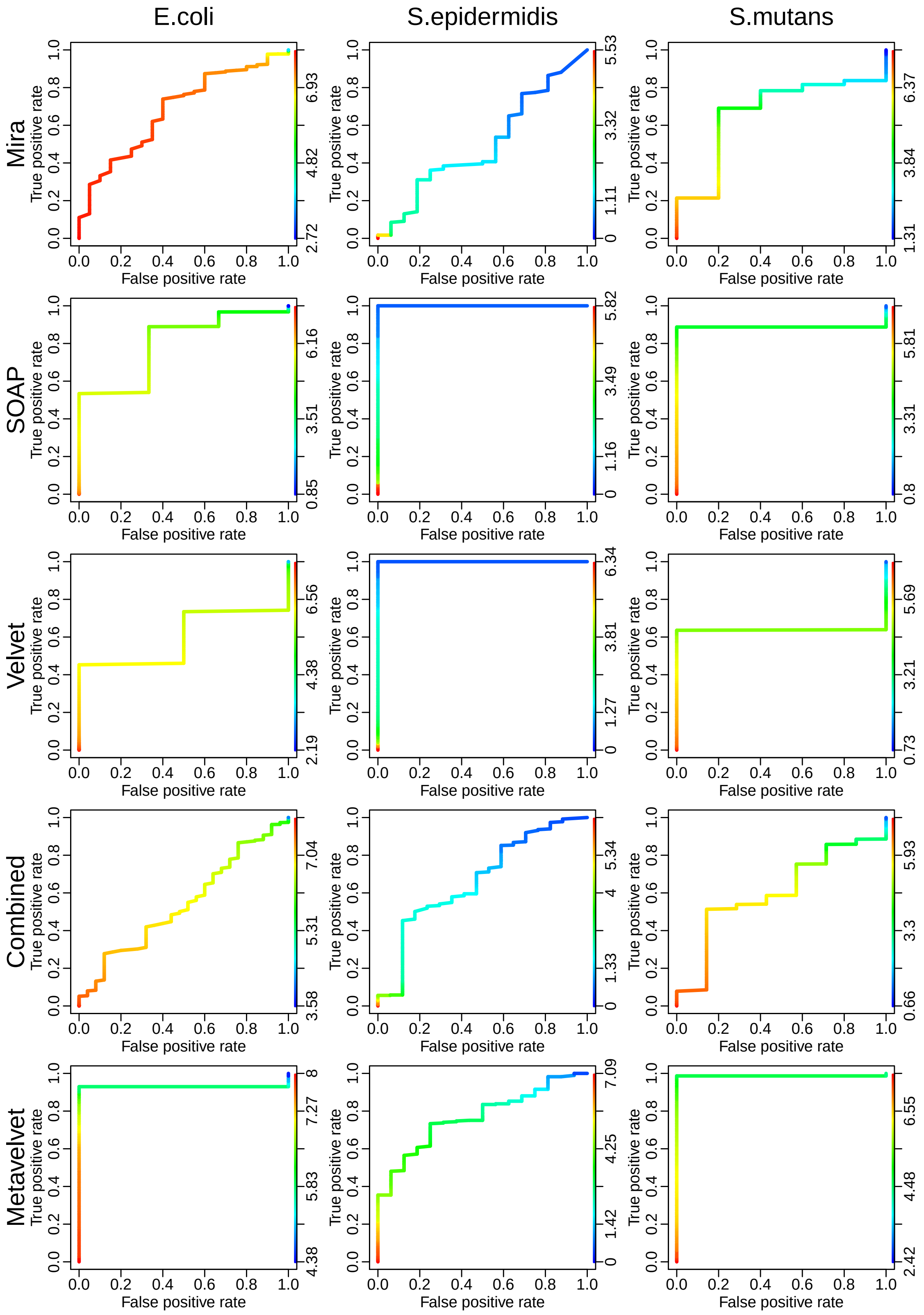

Figure 4: Evaluation of the SuRankCo predictions of major organisms in the mock community test data. The results of the mixed prediction sets are separatly illustrated for single organisms. Each plot comprises a ROC curve of the contig evaluation score grouping in contrast to a varying grouping of the SuRankCo scores. Thereby, the changing color of the graph represents the changing threshold for the SuRankCo score grouping.

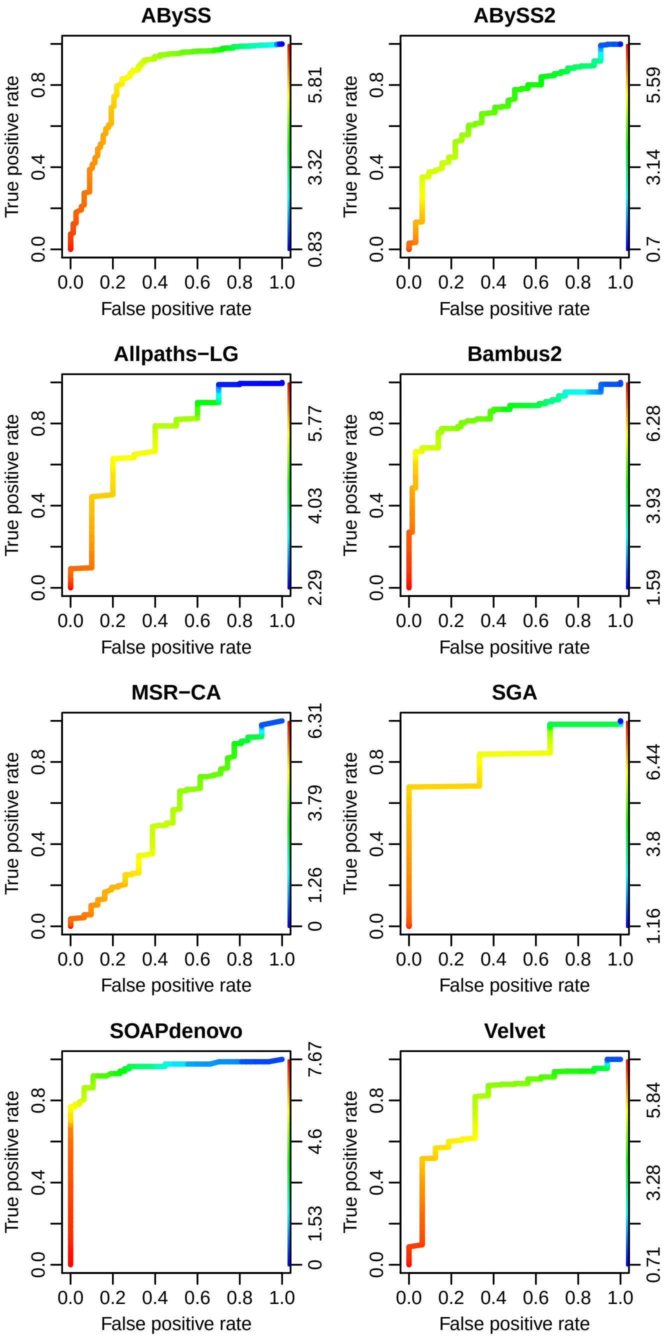

Figure 5: Evaluation of the SuRankCo predictions of the GAGE assemblies. Here, one ROC curve represents the evaluation of *R. sphaeroidis* assemblies classified by the single training dataset. Each plot comprises a ROC curve of the contig evaluation score grouping in contrast to a varying grouping of the SuRankCo scores. Thereby, the changing color of the graph represents the changing threshold for the SuRankCo score grouping.

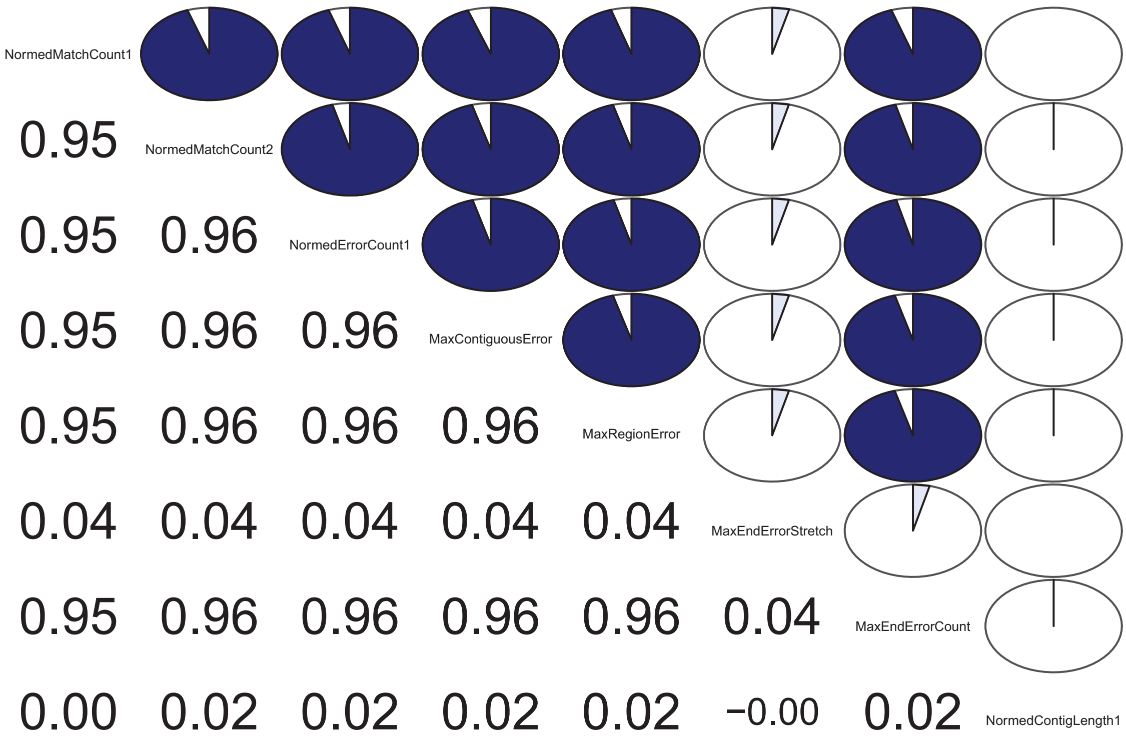

Figure 6: Correlation of single score predictions. The predictions of the single scores for the *E. coli* test data are highly correlated except for the Max End Error Stretch and Normed Contig Length 1. In general, contig score predictions may be less correlated since the contigs used in training may have a lower quality with higher variance in their alignments to the reference sequence.

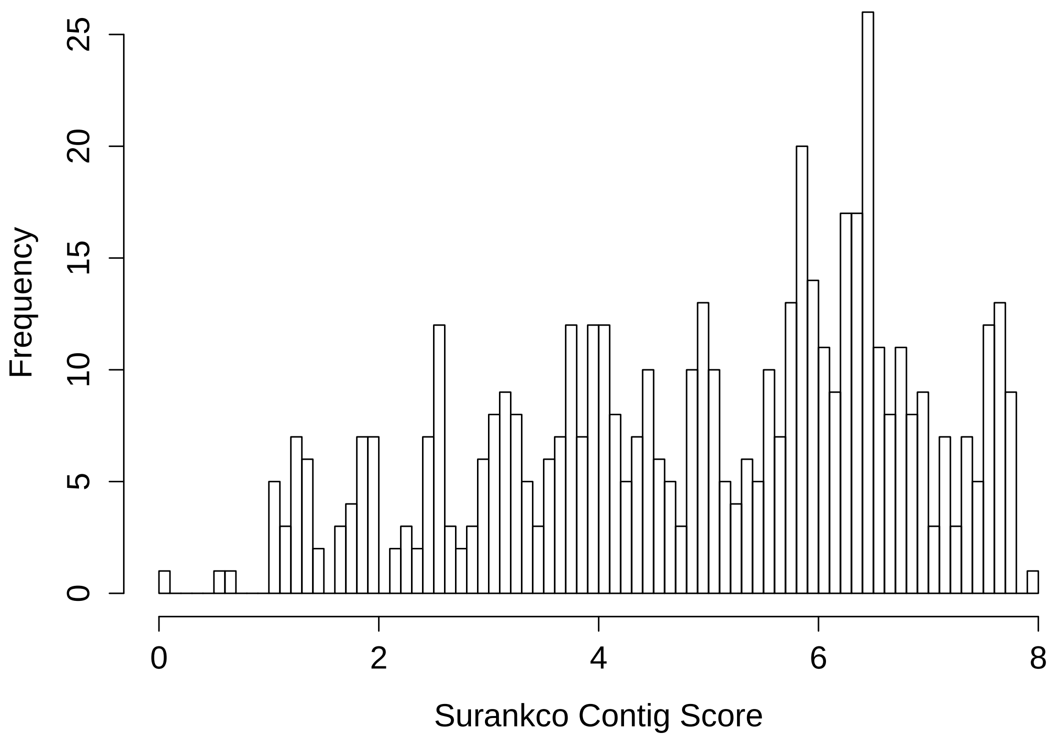

Figure 7: Example histogram of the final SuRankCo score. The histogram is constructed for the prediction set of meta-assembled mock community data. It shows a broad distribution of scores in contrast to the clustered scores of the *E. coli* experiment.

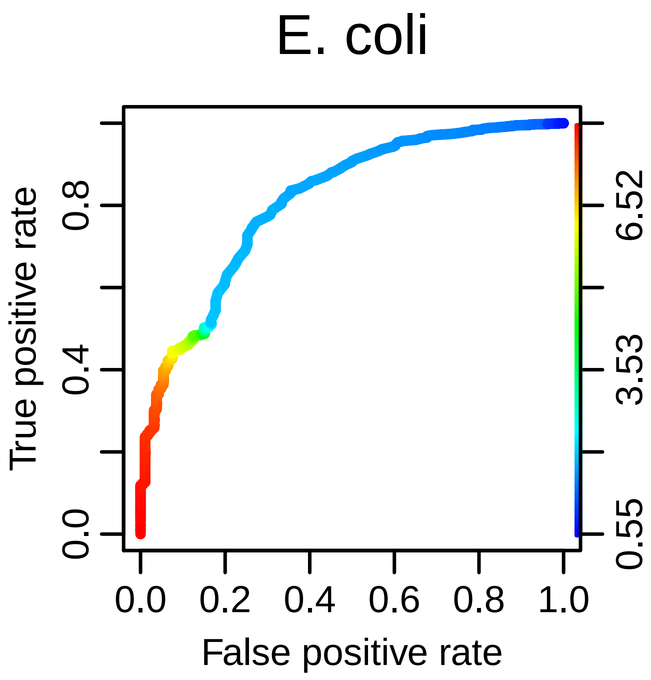

Figure 8: Evaluation of the SuRankCo predictions of the *E. coli* assembly. For completeness and comparability, this figure features the ROC curve-based evaluation of the E. coli experiment as applied for the mock and GAGE experiments. The ROC curve is based on the contig evaluation score grouping in contrast to a varying grouping of the SuRankCo scores. Thereby, the changing color of the graph represents the changing threshold for the SuRankCo score grouping.

S6 Additional Result Tables

Table 4: Organisms of the mock community data, their grouping for the experiments (T and P), assigned contigs of the meta-assembly and the used references.

Organism	Group	N. Ctgs	Accession N.
Acinetobacter baumannii ATCC 17978	T	0	NC_009085.1, NC_009084.1, NC_009083.1
Actinomyces odontolyticus F0309	T	0	NZ_GG753644.1, NZ_GG753643.1, NZ_GG753642.1, NZ_GG753641.1, NZ_GG753640.1, NZ_GG753639.1
Candida albicans SC5314	T	0	NW_139421.1 - NW_139833.1 (413 sequences)
Enterococcus faecalis V583	T	0	NC_004668.1, NC_004671.1, NC_004670.1, NC_004669.1
Lactobacillus gasseri ATCC 33323	T	0	NC_008530.1
Methanobrevibacter smithii ATCC 35061	T	500	NC_009515.1
Propionibacterium acnes KPA171202	T	0	NC_006085.1
Rhodobacter sphaeroides 2.4.1	T	953	NC_007493.2, NC_007494.2, NC_007488.2, NC_007489.1, NC_007490.2, NC_009007.1, NC_009008.1
Staphylococcus aureus subsp. aureus N315	T	332	NC_002745.2, NC_003140.1
Streptococcus agalactiae 2603V-R	T	0	NC_004116.1
Streptococcus pneumoniae R6	T	0	NC_003098.1
Bacillus cereus ATCC 10987	P	0	NC_003909.8, NC_005707.1
Bacteroides vulgatus ATCC 8482	P	0	NC_009614.1
Clostridium beijerinckii NCIMB 8052	P	3	NC_009617.1
Deinococcus radiodurans R1	P	0	NC_001264.1, NC_001263.1, NC_000959.1, NC_000958.1
Escherichia coli str. K-12 substr. MG1655	P	72	NC_000913.3
Helicobacter pylori 26695	P	0	NC_000915.1
Listeria monocytogenes EGD-e	P	0	NC_003210.1
Neisseria meningitidis MC58	P	1	NC_003112.2
Pseudomonas aeruginosa PAO1	P	7	NC_002516.2
Staphylococcus epidermidis ATCC 12228	P	301	NC_004461.1, NC_005003.1, NC_005004.1, NC_005005.1, NC_005006.1, NC_005007.1, NC_005008.1
Streptococcus mutans UA159	P	151	NC_004350.2

Table 5: Spearman correlation between SuRankCo Contig Scores and BLAT metrics of the mock community meta-assembly prediction set.

	**mismatch**	**Qgapcount**	**Tgapcount**	**blockcount**
NormedMatchCount1	-0.8028354	-0.4541743	-0.4047294	-0.4354852
NormedMatchCount2	-0.7737683	-0.4533330	-0.5261784	-0.5462121
NormedErrorCount1	0.7736162	0.4536809	0.5270433	0.5469730
MaxContiguousError	0.7213388	0.4067623	0.4932727	0.5030182
MaxRegionError	0.7496859	0.4630718	0.5411223	0.5592339
MaxEndErrorStretch	0.1095816	0.1295148	0.1097870	0.1188257
MaxEndErrorCount	0.5545051	0.3505190	0.4133438	0.4269306
NormedContigLength1	-0.4434447	-0.7340355	-0.9966364	-0.9461041

Table 6: Spearman correlation between SuRankCo Scores and BLAT metrics of the *E. coli* experiment.

	**mismatch**	**Qgapcount**	**Tgapcount**	**blockcount**
NormedMatchCount1	-0.3865286	-0.0510903	0.1895618	0.1775827
NormedMatchCount2	-0.26312242	-0.05414976	-0.29729808	-0.29618498
NormedErrorCount1	0.26249300	0.05421344	0.29948468	0.29830996
MaxContiguousError	0.21822948	0.04766507	0.29486387	0.29224683
MaxRegionError	0.3624949	0.0614517	0.3318000	0.3309938
MaxEndErrorStretch	-0.33852832	0.03047685	-0.05786300	-0.05406256
MaxEndErrorCount	0.36263360	0.06136507	0.33169810	0.33089688
NormedContigLength1	0.19519882	-0.09248936	-0.99966452	-0.97335101

Table 7: Organism relationships based on reads, calculated by a sub-method of the GASiC tool (Lindner and Renard, 2012)

	E.coli	*M.smithii*	*R.sphaeroides*	*S.aureus*	*S.epidermidis*	*S.mutans*
E.coli	1	0	0.0003	0.000105	0.000556	0.000556
M.smithii	0	1	0	0	0	0
R.sphaeroides	0.000736	0	1	0.000065	0.00044	0.000651
S.aureus	0.0004	0	0.000155	1	0.042757	0.003227
S.epidermidis	0.00041	0	0.00015	0.028878	1	0.003173
S.mutans	0.000325	0	0.00014	0.000546	0.002463	1

References

Altschul,S. *et al.* (1990) Basic local alignment search tool. J Mol Biol, 215:403–410.

Chevreux,B. *et al.* (1999) Genome Sequence Assembly Using Trace Signals and Additional Sequence Information. Computer Science and Biology: Proceedings of the German Conference on Bioinformatics (GCB) 99, ed. Wingender E (GBF-Braunschweig, Hannover, Germany), pp 45–56.

Langmead, B. and Salzberg,S.L. (2012) Fast gapped-read alignment with Bowtie 2. Nat Methods 2012, 9:357–359.

Lindner,M. and Renard, B. (2012) Metagenomic abundance estimation and diagnostic testing on species level. Nucleic Acids Res., 41(1), e10.

Luo,R. *et al.* (2012) SOAPdenovo2: an empirically improved memory-efficient short-read de novo assembler. Gigascience, 1,18.

Namiki,T. *et al.* (2012) MetaVelvet: an extension of Velvet assembler to de novo metagenome assembly from short sequence reads. Nucleic Acids Research, 40(20):e155–e155

Treangen,T.J. *et al.*(2011) Next generation sequence assembly with AMOS. Curr Protoc Bioinformatics Chapter 11: Unit 11 18.

Venables,W.N. and Ripley,B.D. (2002) Modern Applied Statistics with S. Fourth Edition. Springer, New York. ISBN 0-387-95457-0

Wheeler,D.L. *et al.* (2008) Database resources of the National Center for Biotechnology Information. Nucleic Acids Research, 36, D13–21.

Zerbino, D.R. and Birney, E (2008) Velvet: algorithms for de novo short read assembly using de Bruijn graphs. Genome research, 5, 821–9
